# Putative mitochondrial α-ketoglutarate-dependent dioxygenase Fmp12 controls utilization of proline as an energy source in *Saccharomyces cerevisiae*

**DOI:** 10.15698/mic2016.10.535

**Published:** 2016-09-19

**Authors:** Ikuhisa Nishida, Daisuke Watanabe, Hiroshi Takagi

**Affiliations:** 1 Graduate School of Biological Sciences, Nara Institute of Science and Technology, 8916-5 Takayama, Ikoma, Nara 630-0192, Japan.

**Keywords:** yeast Saccharomyces cerevisiae, proline, carbon source, Fmp12, mitochondria, α-ketoglutarate-dependent dioxygenase

## Abstract

The amino acid proline functions as a nitrogen source and as a stress protectant in the yeast *Saccharomyces cerevisiae*. However, utilization of proline as a carbon source in *S. cerevisiae* cells has not been studied yet. In the process of study on the physiological roles of the found-in-mitochondrial-proteome (*FMP*) genes in proline metabolism, we found that Δ*fmp12* cells could grow better than wild-type cells on agar plate medium containing proline as the sole nitrogen and carbon sources. In contrast, overexpression of *FMP12* negatively affected cell growth under the same condition. The Fmp12 protein was localized in the mitochondria and was constitutively expressed. Deletion of the genes that encode mitochondrial enzymes, such as proline dehydrogenase (*PUT1*), Δ^1^-pyrroline-5-carboxylate dehydrogenase (*PUT2*), alanine transaminase (*ALT1*), and α-ketoglutarate dehydrogenase subunit (*KGD1*), abolished the enhanced cell growth in Δ*fmp12*. These results provided the first evidence that proline can be utilized as a carbon source via the mitochondrial proline metabolic pathway and the subsequent tricarboxylic acid (TCA) cycle in *S. cerevisiae*. The function of Fmp12, which has a similarity with α-ketoglutarate-dependent dioxygenases of the yeast *Candida* species and human, might inhibit cell growth by skipping the ATP production step of the TCA cycle.

## INTRODUCTION

Proline is an important amino acid that is used not only for a nitrogen source but also a stress protectant in the budding yeast *Saccharomyces cerevisiae* 1. Therefore, the metabolic regulation of proline, including biosynthesis, degradation, and cellular localization, has been of great interest. *S. cerevisiae* cells synthesize proline from glutamate by three cytoplasmic enzymes, the γ-glutamyl kinase Pro1, the γ-glutamyl phosphate reductase Pro2, and the Δ^1^-pyrroline-5-carboxylate (P5C) reductase Pro3 2. In most of eukaryote cells, including *S. cerevisiae*, proline is degraded predominantly in mitochondria, although the responsible import mechanism into mitochondria has not been clarified yet. The mitochondrial proline dehydrogenase Put1 converts proline into P5C, which is then processed into glutamate by the P5C dehydrogenase Put2 3. As glutamate can be converted into α-ketoglutarate, a tricarboxylic acid (TCA)-cycle intermediate, by deamination or transamination 4,5, it is presumed that proline can be used as a carbon source for ATP production. Nevertheless, there have been few reports on the growth of *S. cerevisiae* cells in the presence of proline or the other amino acids as the sole carbon source. In contrast, several kinds of bacteria and xylose-fermenting yeast *Scheffersomyces stipitis* have been reported to grow by utilization of amino acids as the sole carbon source via glutamate dehydrogenase or amino acid transaminase (AAT) 6,7,8,9. Based on these facts, *S. cerevisiae* cells might possess an unknown metabolic regulatory mechanism that restricts the consumption of amino acids as carbon sources.

To find novel regulators of intracellular proline, we previously performed a transcriptomic analysis of the *S. cerevisiae* *put1* mutant in response to exogenous proline 10. As a result, the accumulation of intracellular proline led to downregulation of the proline-synthetic genes and upregulation of the genes that encode the proline-degradative enzymes, the known proline permeases on the plasma membrane, and the Avt proteins associated with bidirectional transport of proline across the vacuolar membrane. In this study, we analyzed another class of the upregulated genes that encode poorly-understood proteins originally found in the mitochondrial proteome analyses 11,12, because these genes (the found-in-mitochondrial-proteome (*FMP*) genes) and their products (the Fmp proteins) might be involved in novel regulatory mechanisms of proline localization or metabolism in the mitochondria.

## RESULTS

**Figure 1:**
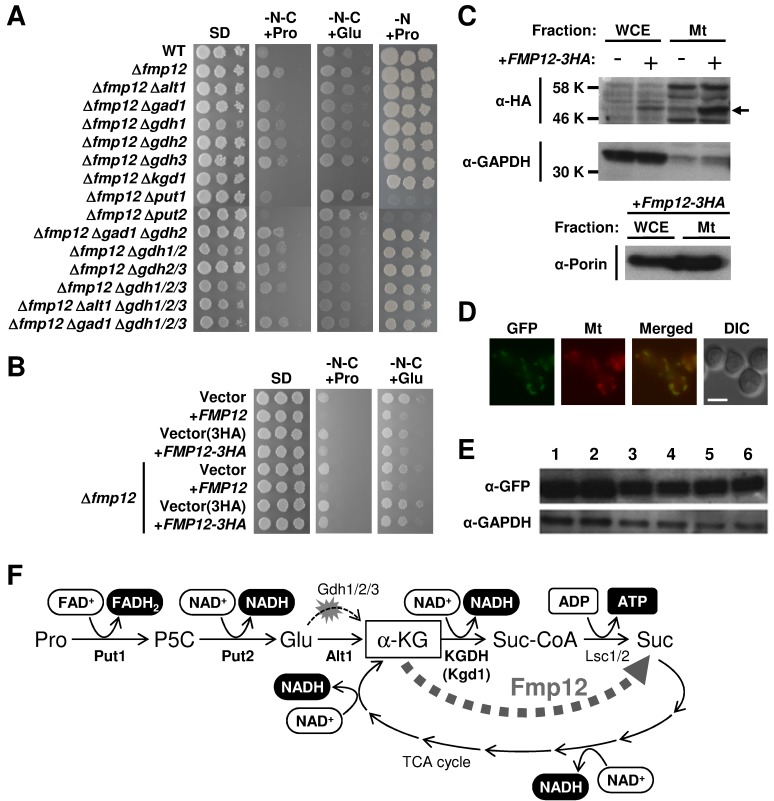
FIGURE 1: Proline/glutamate metabolism as the sole carbon source and its control mediated by Fmp12. **(A)** Growth phenotypes of *S. cerevisiae* wild-type strain (BY4741) and its deletion mutants on SD, SD-N-C+Pro, SD-N-C+Glu, and SD-N+Pro media. **(B) **Growth phenotypes of *S. cerevisiae* BY4741u or BY4741u Δ*fmp12* cells harboring the empty vector (pVV208, pVV209) or overexpressing *FMP12* (pVV208-FMP12, pVV209-FMP12) on SD, SD-N-C+Pro, and SD-N-C+Glu media. ** (C)** Detection of Fmp12-3HA in the whole cell extract (WCE) and in the mitochondria fraction (Mt). The symbol -/+ indicates the sample from the cells without or with expression of Fmp12-3HA from pVV209-FMP12. The arrow indicates the predicted size of Fmp12-3HA truncated at its amino-terminal MTS. GAPDH and porin were analyzed as controls of cytoplasmic and mitochondrial proteins, respectively. **(D)** Subcellular localization of Fmp12-yeGFP by fluorescent microscopy. GFP signals (GFP), MitoTracker signals (Mt), merged signals (Merged), and differential interference contrast images (DIC) are shown. The scale bar represents 5 μm. ** (E) **Western blot analysis of Fmp12-yeGFP under different nutritional conditions. *S. cerevisiae* *FMP12-yeGFP* cells were cultivated on solid media at 30°C for 5 days. The numbers indicate the media as follows: 1: SD, 2: SD+Pro, 3: SD-N-C+Pro, 4: SD-C+Pro, 5: SD-N-C+Glu, 6: SD-C+Glu. GAPDH was used as a protein-loading control. **(F) **Energy production by proline metabolism as the sole carbon source in *S. cerevisiae*. Pro: proline, P5C: Δ^1^-pyrroline-5-carboxylate, Glu: glutamate, α-KG: α-ketoglutarate, Suc-CoA: succinyl-CoA, Suc: succinate.

To examine cell growth in the presence of proline as the sole carbon and nitrogen sources, we used minimal synthetic-defined (SD) medium without ammonium sulfate and glucose and with proline (SD-N-C+Pro). Under such a severe nutrient condition, BY4741 wild-type cells in which the auxotrophic mutations were complemented could only slightly grow during 7-day incubation (Figure 1A). Interestingly, among the *FMP* genes (*FMP12/16/21/27/33/41/45/46/48/52*) that were upregulated by exogenous proline in our previous study 10, deletion of the *FMP12/AIM17* gene was found to markedly enhance the growth on SD-N-C+Pro agar plates (Figure 1A), as well as on SD-C+Pro agar plates, in which proline and ammonium sulfate was added as the sole carbon and nitrogen source, respectively (data not shown). In contrast, overexpression of the *FMP12* gene inhibited the growth on SD-N-C+Pro agar plates (Figure 1B). Similar results were obtained from monosodium glutamate as the sole carbon and nitrogen sources (SD-N-C+Glu) instead of proline, although yeast cells grew better in SD-N-C+Glu than in SD-N-C+Pro. These data suggest the presence of the proline/glutamate metabolic pathway as the sole carbon source in *S. cerevisiae*, although this pathway might be strictly restricted by an unknown mechanism involving Fmp12.

Fmp12 was originally identified in high-throughput mitochondrial proteome analyses 11,12. To determine the subcellular localization of Fmp12, we used HA- or yeGFP-tagged Fmp12 at the carboxyl terminus. Overexpression of Fmp12-3HA was shown to inhibit cell growth on SD-N-C+Pro or SD-N-C+Glu medium (Figure 1B), suggesting that carboxyl-terminus tagged Fmp12 proteins partly functions. It is also noted that the growth of Fmp12-yeGFP-expressing cells on SD-N-C+Pro or SD-N-C+Glu medium was comparable to that of wild-type cells (data not shown). In a western blot analysis (Figure 1C), the HA signals dependent on overexpression of Fmp12-3HA were detected at the lower molecular weight than we expected (approximately 58 kDa) both in the whole cell extract and in the mitochondrial fraction. This is probably because amino-terminal 46 amino acid residues, which correspond to a mitochondrial-targeting signal (MTS) predicted by MitoProt II analysis (https://ihg.gsf.de/ihg/mitoprot.html), were truncated 13. As shown in Figure 1C, a stronger signal of Fmp12-3HA was observed in the mitochondria fraction, which was indicated by a lower level of the cytoplasmic glyceraldehyde-3-phosphate dehydrogenase (GAPDH) and a higher level of the mitochondrial porin protein, than in the whole cell extract. Additionally, the fluorescence microscope observation revealed that Fmp12-yeGFP-derived fluorescent signals colocalized with the MitoTracker signals (Figure 1D). Based on these results, it was concluded that Fmp12 specifically localizes at the mitochondria. The expression level of Fmp12-yeGFP was not significantly affected by exogenous carbon or nitrogen sources (Figure 1E), although the *FMP12* mRNA was previously found to be upregulated by the addition of proline to the medium 10.

To identify the metabolic pathway of proline as a carbon source in Δ*fmp12* cells, we examined the effects of gene deletions on the growth on SD-N-C+Pro medium (Figure 1A). Enhanced growth of Δ*fmp12* cells was almost fully suppressed by a single disruption of the *PUT1* (encoding proline dehydrogenase), *PUT2 *(encoding P5C dehydrogenase), *ALT1* (encoding mitochondrial alanine transaminase) 14, or *KGD1* (encoding E1 component of the mitochondrial α-ketoglutarate dehydrogenase (KGDH) complex) 15 gene, but not affected by single or combined deletion of the *GDH2* (encoding NAD^+^-dependent glutamate dehydrogenase) and *GDH1/3* (encoding NADP^+^-dependent glutamate dehydrogenases) genes involved in deamination of glutamate and its reverse reaction, respectively 5,16,17, or by the *GAD1* (encoding glutamate decarboxylase) gene, whose product catalyzes the first step of the γ-aminobutyrate (GABA) shunt 18 that bypasses the TCA cycle. Similar results were obtained on SD-N-C+Glu medium, although disruption of the *PUT1* or *PUT2* gene did not abolish the growth of Δ*fmp12* cells. Thus, it was demonstrated that utilization of proline as a carbon source is specifically carried out by mitochondrial enzymes Put1 and Put2 for conversion into glutamate, by mitochondrial AATs, such as the alanine transaminase Alt1, for conversion of glutamate into α-ketoglutarate, and subsequently by the TCA cycle (Figure 1F). Since Put1 and Put2, but not Alt1 and Kgd1, are necessary for utilization of proline as a nitrogen source judging from the growth on SD-N+Pro medium (Figure 1A), AATs and KGDH were identified as specific factors required for utilization of proline as the sole carbon source.

## DISCUSSION

Compartmentalization of metabolic enzymes might contribute to optimization of the corresponding metabolic efficiency. If the enzymes with different subcellular localizations are engaged in a metabolic pathway, intermediate compounds must be properly translocated to the sites or the organelles in advance to individual reactions. Although cells might adopt such spatial regulations to constitute highly elaborate metabolic networks, enforcement of a single metabolic pathway is likely achieved by collecting all related enzymes together in a single intracellular area. For instance, it was recently reported that artificial overexpression of the Ehrlich pathway enzymes in mitochondria led to a striking increase in the fusel alcohol production in *S. cerevisiae* 19.

In this study, we discovered a novel proline metabolic pathway essentially mediated by the mitochondrial enzymes Put1, Put2, Alt1, KGDH, and TCA-cycle enzymes (Figure 1F). This compartmentalization might maximize cellular energy production from proline under nutrient-poor environments. The major problem to be solved is how proline is imported into mitochondria. Effects of the mitochondrial transporters on subcellular distribution of proline should be biochemically analyzed in future. It is also noteworthy that the mitochondrial alanine transaminase Alt1 is used for the conversion of glutamate into α-ketoglutarate in *S. cerevisiae*, while glutamate dehydrogenases are responsible in known proline utilization pathways of other yeast species 8. Proline utilization via the alanine transaminase activity has been reported only in parasitic protozoan *Trypanosoma brucei* 20 and tsetse fly 21, which might exhibit similar physiological and nutritional conditions as* S. cerevisiae* cells when proline is the primary carbon source.

To understand how the deletion of *FMP12* enhances cell growth on SD-N-C+Pro medium, we further focused on its molecular function. The Fmp12 protein shows a marked similarity to α-ketoglutarate-dependent dioxygenases conserved from bacteria to human 22, such as the pathogenic yeast *Candida albicans *γ-butyrobetaine dioxygenase Bbh1 and trimethyllysine dioxygenase Bbh2 mediating the carnitine biosynthesis pathway 23. They commonly couple decarboxylation of α-ketoglutarate into succinate to hydroxylation of numerous kinds of cosubstrates (*e.g.* γ-butyrobetaine and trimethyllysine). Although the specific substrate for Fmp12 has not been known yet, the Fmp12-dependent decarboxylation of α-ketoglutarate might enable to bypass the TCA-cycle reactions mediated by KGDH (from α-ketoglutarate to succinyl-CoA) and succinyl-CoA ligase (from succinyl-CoA to succinate) (Figure 1F). If α-ketoglutarate prefers Fmp12 to KGDH, however, NADH production via KGDH and ATP production via succinyl-CoA ligase might be skipped. In particular, when proline is the sole carbon source, the only substrate-level phosphorylation process for ATP production might be impaired by the Fmp12 activity. Therefore, the deletion of *FMP12* might enhance energy production and cell growth on SD-N-C+Pro medium. To prove this hypothesis, the affinity of Fmp12 or KGDH for α-ketoglutarate should be enzymatically determined. If this is the case, it could be concluded that Fmp12 plays a critical inhibitory role in utilization of amino acids as a carbon source. Nutrient preferences of *S. cerevisiae* cells are strictly determined by several mechanisms including carbon/nitrogen catabolite repression 24,25. Analysis of this novel Fmp12 protein will accelerate the understanding of the coordinated mechanism of nutrient sensing and growth control in *S. cerevisiae*.

## MATERIALS AND METHODS

### Strains

Yeast strains used in this study were the *S. cerevisiae* strains with a BY4741 (*MAT***a ***his3*Δ*1 leu2*Δ*0 met15*Δ*0 ura3*Δ*0*) background (provided by Open Biosystems or National Bio-Resource Project (NBRP) of the MEXT, Japan) and BY4741u (*MAT***a ***ura3*Δ*0*) background (constructed by S. Morigasaki). The yeast strains used in this study are listed in Table 1. In strain BY4741, the original genes on the chromosomes were disrupted by a standard PCR-based gene disruption or tagging method. Disruption cassettes amplified by PCR with pFA6a-GFP(S65T)-His3MX6 (Addgene), pFA6a-hphNT1 (Euroscarf), pFA6a-natNT2 (Euroscarf), or an S1-AUR1-S2 fragment derived from pAUR123 (Takara Bio) using gene-specific oligonucleotide primers were integrated into each locus of the target genes. Fmp12 was carboxyl terminally-tagged with yeGFP using pYM25 (Euroscarf). Gene disruptants were selected on YPD medium containing G418 (200 μg/ml), clonNAT (100 μg/ml), hygromycin B (300 μg/ml), or aureobasidin A (0.25 μg/ml). SC-His medium was used to obtain strains disrupted with *CgHIS3* containing disruption cassettes. Correct disruption was confirmed by colony PCR using up and down primers of the target genes. When appropriate, required empty vectors pAD4 (provided by J. Nikawa), pRS416MET15 (constructed by S. Morigasaki), pRS416CgHIS3MET15 (constructed by S. Morigasaki) were introduced to complement the auxotrophy. *E. coli* strains DH5α (F^-^λ^-^Φ*80lacZ*Δ*M15* Δ(*lacZYA argF*)*U169 deoR recA1 endA1 hsdR17*(*r_k_*^-^*m_k_*^+^) *supE44 thi-1 gyrA96*) and DB3.1 (F^-^* gyrA462 endA1* Δ(sr1-*rec*A) *mcr*B *mrr hsdS20*(r_B_^-^ m_B_^-^) *supE44* *ara*-*14* *galK2* *lacY1* *proA2 rpsL20*(SmR) *xyl*-5 λ- *leu mtl1*) were used for construction of the plasmids.

**Table 1:** Yeast strains in this study.

**Strain**	**Genotype**
BY4741	*MAT***a** *his3*Δ*1* *leu2*Δ*0 met15*Δ*0 ura3*Δ*0*
Δ*fmp12*	BY4741 Δ*fmp12::kanMX4*
Δ*fmp12* Δ*alt1*	BY4741 Δ*fmp12::kanMX4 *Δ*alt1::hphNT1*
Δ*fmp12* Δ*gad1*	BY4741 Δ*fmp12::kanMX4 *Δ*gad1::hphNT1*
Δ*fmp12* Δ*gdh1*	BY4741 Δ*fmp12::kanMX4 *Δ*gdh1::hphNT1*
Δ*fmp12 *Δ*gdh2*	BY4741 Δ*fmp12::kanMX4 *Δ*gdh2::hphNT1*
Δ*fmp12* Δ*gdh3*	BY4741 Δ*fmp12::kanMX4 *Δ*gdh3::hphNT1*
Δ*fmp12* Δ*kgd1*	BY4741 Δ*fmp12::kanMX4 *Δ*kgd1::hphNT1*
Δ*fmp12* Δ*put1*	BY4741 Δ*fmp12::kanMX4 *Δ*put1::hphNT1*
Δ*fmp12* Δ*put2*	BY4741 Δ*fmp12::kanMX4 *Δ*put2::hphNT1*
Δ*fmp12 *Δ*gad1 *Δ*gdh2*	BY4741 Δ*fmp12::kanMX4 *Δ*gad1::hphNT1 *Δ*gdh2::natNT2*
Δ*fmp12* Δ*gdh1/2*	BY4741 Δ*fmp12::kanMX4 *Δ*gdh1::hphNT1 *Δ*gdh2::natNT2*
Δ*fmp12* Δ*gdh2/3*	BY4741 Δ*fmp12::kanMX4 *Δ*gdh2::natNT2 *Δ*gdh3::hphNT1*
Δ*fmp12* Δ*gdh1/2/3*	BY4741 Δ*fmp12::kanMX4 *Δ*gdh1::AUR1-C *Δ*gdh2::natNT2 *Δ*gdh3::hphNT1*
Δ*fmp12* Δ*alt1* Δ*gdh1/2/3*	BY4741 Δ*fmp12::kanMX4 *Δ*alt1::HISMX6 *Δ*gdh1::AUR1-C *Δ*gdh2::natNT2 *Δ*gdh3::hphNT1*
Δ*fmp12* Δ*gad1* Δ*gdh1/2/3*	BY4741 Δ*fmp12::kanMX4 *Δ*gad1::HISMX6 *Δ*gdh1::AUR1-C *Δ*gdh2::natNT2 *Δ*gdh3::hphNT1*
*Fmp12-yeGFP*	BY4741 *FMP12::FMP12-yeGFP-hphNT1*
BY4741u	*MAT***a** *ura3*Δ*0*
BY4741u Δ*fmp12*	*MAT***a** *ura3*Δ*0 *Δ*fmp12::kanMX4*

### Plasmids

For overexpression of Fmp12, we constructed plasmids containing a Tet-Off system 26. BP and LR recombination reaction procedures were performed as recommended by Invitrogen (on the Internet at www.invitrogen.com). Plasmid BG1805-FMP12 (Addgene) containing oligonucleotides of *FMP12* without stop codon flanked with attB1 or attB2 sequences was cloned into pDONR221 (Invitrogen) using the BP reaction. The cloned fragment was then inserted into the empty vector pVV208 (*CEN URA3 pTetO7*) or pVV209 (*CEN URA3 pTetO7 3HA*) 26 by the LR reaction.

### Culture Media

The media used for growth of *S. cerevisiae* were a nutrient medium YPD (1% yeast extract, 2% peptone, and 2% glucose) and a synthetic minimal medium SD (0.17% yeast nitrogen base without amino acids and ammonium sulfate (Difco Laboratories), 0.5% ammonium sulfate, and 2% glucose). When appropriate, 3% proline or 3% monosodium glutamate were added to the media as the sole carbon and nitrogen source. For SD-N+Pro medium, 0.1% proline was added instead of ammonium. If necessary, 2% agar was added to solidify the medium. All amino acids used in this study are l-form. *E. coli* cells were grown in Luria-Bertani (LB) complete medium (0.5% Bacto yeast extract, 1% Bacto tryptone (Difco Laboratories), 1% NaCl, pH 7.0) with 100 μg/ml ampicillin or 50 μg/ml kanamycin.

### Growth Test

Fresh yeast cells were cultured in SD liquid medium for 1 day at 30°C. After washing the cells with distilled water three times, cells corresponding to an OD_600_ of 2.0 and serial dilution of 10^-1^ to 10^-3^ were spotted onto the agar plates and were incubated at 30°C for several days.

### Fluorescent Microscopy

Yeast cells expressing Fmp12-yeGFP were grown to reach an OD_600_ of 1 at 25°C in SD liquid medium and subjected to 40 nM MitoTracker Orange CMTMRos (Invitrogen) for 30 min to visualize the mitochondria. The cells were then harvested and washed with distilled water. Fmp12-yeGFP and MitoTracker signals were observed under a fluorescence microscope Axiovert 200M (Carl Zeiss). Images were captured with a HBO 100 Microscope Illuminating System (Carl Zeiss) digital camera.

### Western Blotting Analysis

Yeast cells grown on agar plates for 5 days at 30°C were collected, washed with 10% trichloroacetic acid (TCA), resuspended by 100 μl of 10% TCA, and disrupted with 0.5-mm glass beads in a MultiBead Shocker (Yasui Kikai) with seven cycles of running at 2,500 rpm for 60 seconds and pausing for 60 seconds. After adding 200 μl of 10% TCA to the tube, the pellets were separated from the glass beads by centrifugation. The obtained pellets were resuspended in 150 μL of sample buffer (50 mM Tris-HCl (pH 6.8), 2% sodium dodecyl sulfate, 4.5% glycerol, 0.01% bromophenol blue, 0.7 M 2-mercaptoethanol) and 150 μl of 1 M Tris-HCl (pH 8.0) for neutralization. After boiling for 5 min at 95°C, the supernatant was obtained by centrifugation and the protein concentration was measured by using a Bio-Rad protein assay kit (Bio-Rad). The mitochondrial fraction was isolated according to the method described previously 27 with minor modification. In the case of the Fmp12-3HA protein, lysates of yeast cells grown in lactate minimal medium (0.17% Bacto yeast nitrogen base without amino acids and ammonium sulphate (Difco Laboratories), 0.5% ammonium sulphate, 2% dropout mix, 0.05% glucose, 0.05% CaCl_2_, 0.05% NaCl, 0.03% MgCl_2_, 0.1% KH_2_PO_4_, 2% lactate (pH 5.6)) or the mitochondrial fractions were directly mixed with sample buffer and boiled. Approximately 5 μg of total proteins were loaded onto a 10% SDS-polyacrylamide gel and electrophoresed with a constant voltage at 80-120 V. Proteins were transferred to a nitrocellulose membrane (Hybond-C; GE Healthcare) in blotting buffer (48 mM Tris, 385 mM glycine, 0.1% SDS, 20% methanol) (115 V, 400 mA, 1 h). The membrane was soaked in blocking buffer (Tris-buffered saline with 0.3% Tween-20, 3% skim milk, 0.03% sodium azide (pH 8.0)) for more than 1 h. Anti-GFP mouse monoclonal (Roche Diagnostics), anti-HA rabbit polyclonal (Santa Cruz Biotechnology), anti-yeast GAPDH rabbit polyclonal (Nordic Immunology) and anti-yeast porin mouse monoclonal (Invitrogen) antibodies were used as primary antibodies, and horseradish peroxidase-fused anti-mouse or anti-rabbit immunoglobulin G (Promega) were used as a secondary antibody. Detection was performed using the Pierce ECL Plus Western Blotting Substrate (Thermo Fisher).
